# Cancer-specific mortality after radical prostatectomy vs external beam radiotherapy in high-risk Hispanic/Latino prostate cancer patients

**DOI:** 10.1007/s11255-021-03055-7

**Published:** 2021-11-16

**Authors:** Benedikt Hoeh, Jan L. Hohenhorst, Rocco Flammia, Benedikt Horlemann, Gabriele Sorce, Francesco Chierigo, Zhe Tian, Fred Saad, Markus Graefen, Michele Gallucci, Alberto Briganti, Carlo Terrone, Shahrokh F. Shariat, Luis A. Kluth, Andreas Becker, Felix K. H. Chun, Pierre I. Karakiewicz

**Affiliations:** 1 Department of Urology, University Hospital Frankfurt, Goethe University Frankfurt Am Main, Theodor-Stern-Kai 7, 60590 Frankfurt am Main, Germany; 2 Cancer Prognostics and Health Outcomes Unit, Division of Urology, University of Montréal Health Center, Montréal, QC Canada; 3 Martini-Klinik Prostate Cancer Center, University Hospital Hamburg-Eppendorf, Hamburg, Germany; 4 Department of Maternal-Child and Urological Sciences, Policlinico Umberto I Hospital, Sapienza Rome University, Rome, Italy; 5 Division of Experimental Oncology/Unit of Urology, URI, Urological Research Institute, IRCCS San Raffaele Scientific Institute, Milan, Italy; 6 Department of Surgical and Diagnostic Integrated Sciences (DISC), University of Genova, Genova, Italy; 7 Department of Urology, Comprehensive Cancer Center, Medical University of Vienna, Vienna, Austria; 8 Department of Urology, Weill Cornell Medical College, New York, NY USA; 9 Department of Urology, University of Texas Southwestern, Dallas, TX USA; 10 Department of Urology, Second Faculty of Medicine, Charles University, Prague, Czech Republic; 11 Institute for Urology and Reproductive Health, I.M. Sechenov First Moscow State Medical University, Moscow, Russia; 12 Division of Urology, Department of Special Surgery, Jordan University Hospital, The University of Jordan, Amman, Jordan

**Keywords:** High-risk prostate cancer, Radical prostatectomy, External beam radiotherapy, Hispanic–Latino race/ethnicity, Cancer-specific survival

## Abstract

**Purpose:**

To test for differences in cancer-specific mortality (CSM) rates in Hispanic/Latino prostate cancer patients according to treatment type, radical prostatectomy (RP) vs external beam radiotherapy (EBRT).

**Methods:**

Within the Surveillance, Epidemiology, and End Results database (2010–2016), we identified 2290 NCCN (National Comprehensive Cancer Network) high-risk (HR) Hispanic/Latino prostate cancer patients. Of those, 893 (39.0%) were treated with RP vs 1397 (61.0%) with EBRT. First, cumulative incidence plots and competing risks regression models tested for CSM differences after adjustment for other cause mortality (OCM). Second, cumulative incidence plots and competing risks regression models were refitted after 1:1 propensity score matching (according to age, PSA, biopsy Gleason score, cT-stage, cN-stage).

**Results:**

In NCCN HR patients, 5-year CSM rates for RP vs EBRT were 2.4 vs 4.7%, yielding a multivariable hazard ratio of 0.37 (95% CI 0.19–0.73, *p* = 0.004) favoring RP. However, after propensity score matching, the hazard ratio of 0.54 was no longer statistically significant (95% CI 0.21–1.39, *p* = 0.2).

**Conclusion:**

Without the use of strictest adjustment for population differences, NCCN high-risk Hispanic/Latino prostate cancer patients appear to benefit more of RP than EBRT. However, after strictest adjustment for baseline patient and tumor characteristics between RP and EBRT cohorts, the apparent CSM benefit of RP is no longer statistically significant. In consequence, in Hispanic/Latino NCCN high-risk patients, either treatment modality results in similar CSM outcome.

## Introduction

Four smaller-scaled studies did not identify a difference in cancer control rates between radical prostatectomy (RP) vs external beam radiotherapy (EBRT) high-risk localized prostate cancer [1–4]. However, Knipper et al. as well as Chierigo et al. relied on large-scale epidemiological cohorts and did observe better survival after RP vs EBRT in high-risk localized prostate cancer [5, 6]. However, the majority of individuals (70–79%) in those two studies were Caucasian [5, 6]. In line with currently limited specific data on Hispanic/Latino prostate cancer outcomes, we tested the hypothesis that a cancer-specific mortality (CSM) difference may exist between RP vs EBRT in the specific group of Hispanic/Latino high-risk localized prostate cancer patients, who currently represent the second largest race/ethnicity group (19% in 2019) in the USA [7–12]. We addressed this knowledge gap relying on the Surveillance, Epidemiology, and End Results (SEER) database (2010–2016).

## Material and methods

### Study population

The current SEER database samples 34.6% of the US population and approximates it in demographic composition and cancer incidence [13]. Within SEER database 2010–2016, we identified and included all patients ≥ 18 years old with histologically confirmed adenocarcinoma of the prostate, diagnosed at biopsy (International Classification of Disease for Oncology [ICD-O-3] code 8140 site code C61.9) that fulfilled high-risk National Comprehensive Cancer Network (NCCN) prostate cancer criteria (defined as Gleason sum 8–10, or PSA > 20 ng/mL, or clinical stage ≥ T3) [14]. Clinical and pathological staging relied on the adjusted American Joint Committee on Cancer (AJCC) 6th (2010–2015) and AJCC 7th (2016) TNM-classification [15, 16]. Patients with missing vital status, unknown PSA, unknown clinical T-stage/M-stage and unknown biopsy Gleason score were excluded. Moreover, we excluded autopsy- or death certificate-only cases and patients with treatment other than RP or EBRT. CSM was defined as deaths attributable to prostate cancer. Conversely, other cause mortality (OCM) was defined as deaths attributable to other causes than prostate cancer. Follow-up was defined as time from diagnosis to the end of study period, loss to follow-up, CSM or OCM.

### Statistical analyses

Statistical analyses were based on three steps. First, we addressed CSM prior to propensity score matching in NCCN high-risk prostate cancer patients. We relied on cumulative incidence plots to illustrate CSM and competing risks regression models to test for CSM differences, after adjustment for OCM, between RP and EBRT prostate cancer patients. Adjustment covariates consisted of age (year intervals), PSA (in 1 ng/mL intervals), biopsy Gleason score (3 + 3, 3 + 4, 3 + 5, 4 + 3, 4 + 4, 4 + 5, 5 + 3, 5 + 4, 5 + 5), cT-stage (cT1/cT2, cT3a/cT3b/cT4) and cN-stage (cN0, cN1, cNx).

Second, we relied on propensity score matching (PSM). All RP patients were matched in 1:1 fashion to EBRT patients. Matching variables consisted of age (1-year intervals), PSA (in 1 ng/mL intervals), biopsy Gleason score (exact matching), cT-stage (exact matching) and cN-stage (exact matching). After propensity score matching, cumulative incidence plots and competing risks regression models were refitted, using the same covariates as outlined above. For all statistical analyses R software environment for statistical computing and graphics (version 3.4.3) was used. All tests were two-sided with a level of significance set at *p* < 0.05 [17].

## Results

### Descriptive characteristics of the study population

We identified 2290 NCCN high-risk Hispanic/Latino prostate cancer patients. Of those, 893 (39.0%) underwent RP vs 1397 (61.0%) underwent EBRT (Table 1). In general, RP patients were younger (63 vs 70 years; *p* < 0.001), harbored lower PSA values 9 vs 15 ng/mL; *p* < 0.001) and had less aggressive disease stage than EBRT patients (Table 1).

**Table 1:** Descriptive characteristics of 2290 Hispanic/Latino NCCN high-risk prostate cancer patients within the Surveillance, Epidemiology and End Results (2010–2016) database, stratified by treatment type (radical prostatectomy vs external beam radiotherapy) before and after propensity score matching (according to age, PSA, biopsy Gleason score, cT-stage and cN-stage)

	Unmatched data			Propensity score matched data		
	Radical prostatectomy *n* = 893	External beam radiotherapy *n* = 1397	*p*-value	Radical prostatectomy *n* = 524	External beam radiotherapy *n* = 524	*p* value
Age in years, Median (IQR)	63 (57, 68)	70 (64, 75)	< 0.001	66 (61, 70)	65 (61, 70)	0.6
PSA in ng/mL, Median (IQR)	9 (6, 22)	15 (8, 31)	< 0.001	10 (6, 23)	12 (7, 23)	0.2
Biopsy Gleason Score, *n* (%)			< 0.001			0.4
3 + 3	94 (11%)	75 (5.4%)		42 (8.0%)	37 (7.1%)	
3 + 4	105 (12%)	128 (9.2%)		53 (10%)	53 (10%)	
3 + 5	41 (4.6%)	52 (3.7%)		23 (4.4%)	24 (4.6%)	
4 + 3	74 (8.3%)	127 (9.1%)		49 (9.4%)	45 (8.6%)	
4 + 4	363 (41%)	563 (40%)		208 (40%)	235 (45%)	
4 + 5	157 (18%)	298 (21%)		104 (20%)	90 (17%)	
5 + 3	8 (0.9%)	20 (1.4%)		4 (0.8%)	5 (1.0%)	
5 + 4	42 (4.7%)	92 (6.6%)		33 (6.3%)	29 (5.5%)	
5 + 5	9 (1.0%)	42 (3.0%)		8 (1.5%)	6 (1.1%)	
cT-stage, *n* (%)			0.001			0.8
cT1	505 (57%)	739 (53%)		295 (56%)	308 (59%)	
cT2	273 (31%)	492 (35%)		173 (33%)	157 (30%)	
cT3a	71 (8.0%)	71 (5.1%)		30 (5.7%)	34 (6.5%)	
cT3b	40 (4.5%)	76 (5.4%)		23 (4.4%)	23 (4.4%)	
cT4	4 (0.4%)	19 (1.4%)		3 (0.6%)	2 (0.4%)	
cN-stage, *n* (%)			< 0.001			0.1
cN0	781 (87%)	1,336 (96%)		486 (93%)	498 (95%)	
cN1	110 (12%)	42 (3.0%)		37 (7.1%)	23 (4.4%)	
cNx	2 (0.2%)	19 (1.4%)		1 (0.2%)	3 (0.6%)	

*NCCN* National Comprehensive Cancer Network, *PSA* prostate-specific antigen

### Competing risks regression model before propensity score matching

**Fig. 1:**
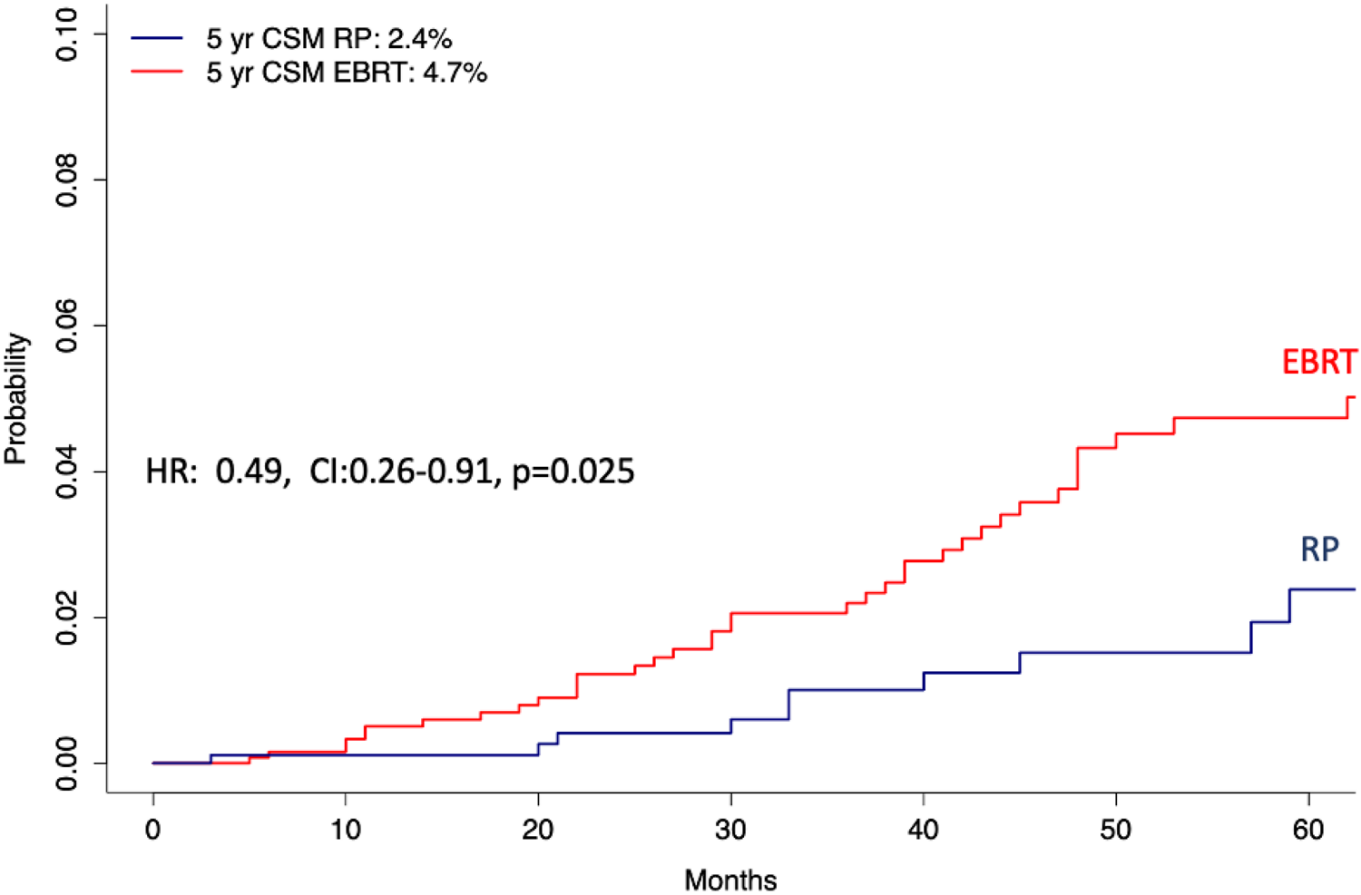
Cumulative incidence plots depicting cancer-specific mortality (CSM) after adjustment for other cause mortality (OCM) in radical prostatectomy vs external beam radiotherapy NCCN high-risk Hispanic/Latino prostate cancer patients. *HR* hazard ratio, *CI* 95%-confidence interval, *NCCN* National Comprehensive Cancer Network

In cumulative incidence models that relied on the entire cohort of 2290 (RP: 893 vs EBRT: 1397) patients, focusing on 5 years of follow-up, CSM rates were 2.4 vs 4.7% (*p* = 0.025) for RP vs EBRT patients, respectively (Fig. 1). This translated into (Table 2) a multivariable competing risks regression hazard ratio of 0.37 (95% CI 0.19–0.73, *p* = 0.004).

**Table 2:** Uni- and multivariable competing risks regression models testing for differences in cancer-specific mortality between radical prostatectomy and external beam radiotherapy prior (*n* = 2290) to and after (*n* = 1048) 1:1 propensity score matching (according to age, PSA, biopsy Gleason score, cT-stage and cN-stage) within the Surveillance, Epidemiology and End Results (2010–2016) database in 2290 NCCN high-risk PCa patients

	Univariable competing risks regression			Multivariable competing risks regression		
	Hazard ratio	95% CI	*p* value	Hazard ratio	95% CI	*p* value
NCCN high-risk						
Unmatched data	0.49	0.26–0.91	0.025	0.37	0.19–0.73	0.004
PSM matched data	0.55	0.20–1.49	0.2	0.54	0.21–1.39	0.2

Matching covariables consisted of age, PSA, biopsy Gleason Score, cT-stage and cN-stage

*NCCN* National Comprehensive Cancer Network, *PSM* propensity score matching, *CI* 95%-confidence interval

### Propensity score matching

One to one propensity score matching was applied to the entire cohort of high-risk patients (*n* = 2290), of whom 893 were treated with RP vs 1397 with EBRT. Propensity score matching resulted in two equally sized groups of 524 RP vs 524 EBRT patients, with no residual statistically significant differences (*p* ≥ 0.1) in patient and tumor characteristics (Table 1).

### Competing risk regression model after propensity score matching

**Fig. 2:**
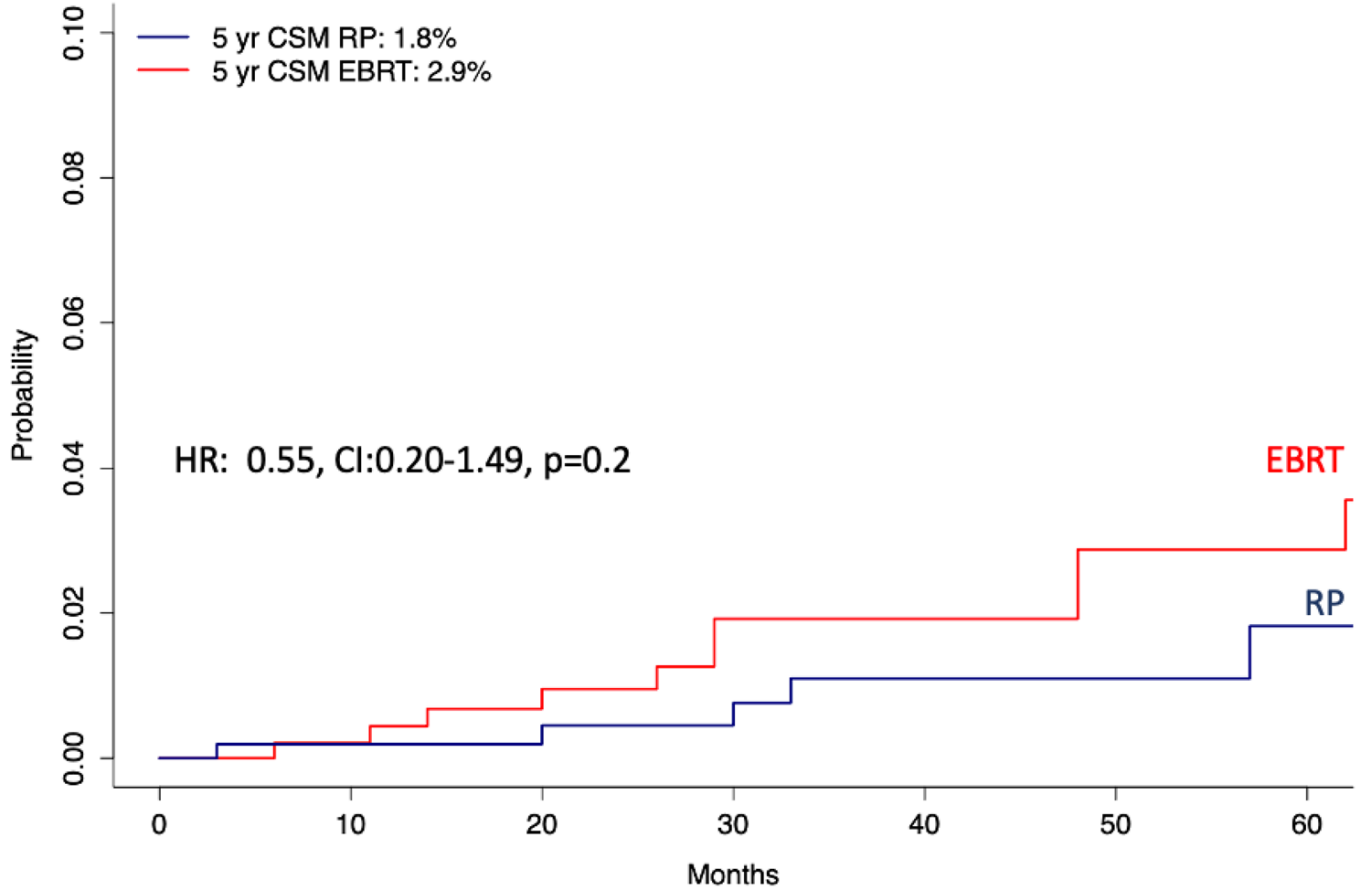
Cumulative incidence plots after 1:1 propensity score matching depicting cancer-specific mortality (CSM) after adjustment for other cause mortality (OCM) in radical prostatectomy (RP) vs external beam radiotherapy (EBRT) NCCN high-risk Hispanic/Latino prostate cancer patients. *HR* hazard ratio, *CI* 95%-confidence interval, *NCCN* National Comprehensive Cancer Network

After propensity score matching, in cumulative incidence models focusing on 5 years of follow-up, CSM rates were 1.9 vs 2.9% (*p* = 0.2) for RP vs EBRT patients, respectively (Fig. 2). This translated into a multivariable competing risk regression hazard ratio of 0.54 (95% CI 0.21–1.39, *p* = 0.2).

## Discussion

Four previous small-scale studies failed to identify a difference in cancer control between RP vs EBRT in high-risk localized prostate cancer patients [1–4]. However, RP patients experienced lower CSM rates than their EBRT counterparts in two contemporary large-scale population-based analyses [5, 6]. However, these two analyses very heavily relied on Caucasian patients. Moreover, stratifications according to race/ethnicity were not performed. In consequence, it is unknown whether this benefit also applies to specific race/ethnicity groups. We tested this hypothesis within the Hispanic/Latino population of NCCN high-risk prostate cancer patients, since Hispanic/Latinos represent the second largest non-Caucasian race/ethnicity group [7].

First, Chierigo et al. [6] reported the proportion of Hispanic/Latino patients within their study, which accounted for 9.4% of the total cohort. This percentage underestimates the proportion of Hispanic/Latino men in the USA, which was 19% in 2019 according to the US (United States) Census Bureau [7]. In consequence, large epidemiological databases such as SEER undersamples Hispanic/Latino and, ideally, Hispanic/Latino patients should be included in larger numbers to provide more meaningful numbers that are better reflective of the true proportion of Hispanic/Latino men in the USA. Although the absolute numbers and proportion of Hispanic/Latino patients could ideally be higher in the SEER database, the latter represents an excellent data pool for analyses addressing differences related to race/ethnicity. It is of interest that the National Cancer Database (NCDB) includes a larger absolute number of Hispanic/Latino patients [18]. However, lack of cancer-specific mortality data within that database renders analyses, such as the current study, impossible to complete using NCDB data.

Second, within Hispanic/Latino prostate cancer patients, we observed very important differences in patient and tumor characteristics. Specifically, on average, EBRT patients were older and presented with more advanced prostate cancer. These differences are very similar in absolute and relative terms to those recorded in analyses that predominantly relied on Caucasian patients. In consequence, the selection criteria for RP vs EBRT that are based on age appear to be very similar between Hispanic/Latino and Caucasian patients [5, 19]. The presence of such differences requires more extensive adjustments than standard multivariable modeling. In consequence, propensity score matching for PSA, biopsy Gleason score, cT-stage and cN-stage, which most closely resembles prospective randomized study design, was applied to maximally control for the effect of residual biases that may persist after standard multivariable adjustment. Finally, differences in OCM that are well established between RP vs EBRT patients and that also apply to Hispanic/Latino patients may further confound analyses of CSM rates [20]. To address these differences, we also relied on competing risks regression models to provide the most unbiased rates of CSM that are adjusted for OCM in addition to standard multivariable adjustment and propensity score matching. Similar methodology was previously applied in comparisons between RP vs EBRT [21].

Third, we tested for CSM differences in NCCN high-risk Hispanic/Latino prostate cancer patients according to treatment, RP vs EBRT. Analyses were first performed without propensity score matching. Subsequently, the same analyses were repeated after propensity score matching. Prior to propensity score matching, RP was associated with lower CSM (multivariable HR: 0.37; 95% CI 0.19–0.73, *p* = 0.004). However, after propensity score matching that reduced the original cohort of 2290 to 1048 highly comparable patients, the CSM benefit of RP was no longer applicable (multivariable HR: 0.54; 95% CI 0.21–1.39, *p* = 0.2). In consequence, after strictest adjustment for population differences as well as for OCM rate differences that exist between RP and EBRT patients, we no longer detected a cancer control benefit of RP over EBRT. Lack of CSM benefit in RP in Hispanic/Latino patients is different from observations made by Knipper et al. as well as Chierigo et al. where a CSM benefit was recorded in the overall group of high-risk patients without stratification according to race/ethnicity. In consequence, it appears that the RP benefit over EBRT treatment is predominantly operational in Caucasian patients. However, it is clearly not operational in Hispanic/Latino patients. Consequently, Hispanic/Latino patients may be expected to benefit equally of either RP or EBRT.

Our study is not devoid of limitations. Our findings originate from an observational cohort and are of retrospective nature. Although we relied on the strictest methodology to maximally reduce biases that are operational between RP and EBRT patients (propensity score matching), as well as competing risks regression methodology that controls for the effect of underlying comorbidities on OCM, our study is not comparable to a prospective randomized design. Even though that strictest adjustment for tumor and patients’ characteristics was applied in the current study, potential residual differences in tumor and patients’ composition cannot be ruled out completely, irrespective of the strictest adjustment methodology. Consequently, results of retrospective population-based studies, such as the current one, should be interpreted accordingly and should be ideally confirmed in prospective randomized trial settings. However, it is unlikely that a prospective, randomized design will ever address CSM differences between Hispanic/Latino NCCN high-risk patients treated either with RP vs EBRT [22, 23]. To date, no data, derived from a randomized clinical trial comparing RP vs EBRT in NCCN high-risk prostate cancer are available. Preliminary results, derived from the only ongoing prospective trial (SPCG-15), comparing RP vs EBRT in NCCN high-risk prostate cancer, will most likely be of insufficient sample size to allow specific subgroup analyses that address Hispanic/Latino patients [24]. In consequence, large-scale population-based analyses, such as the current study, will be required to address smaller race/ethnicity groups such as Hispanic/Latinos or Asian, according to their sample size within epidemiological databases and even more so within prospective randomized trials. It is of note that even though relying on large-scale population-based data repository, such as the SEER, results should still be interpreted under the light of limited sample size and low event rates. Nonetheless, ideally the current results should be validated within a prospective randomized design or tested within an equally large population-based data repository different from the SEER [18].

A potential limitation of the SEER database consists of lack of information regarding comorbidities, which could affect treatment assignment. However, the use of OCM represents an excellent surrogate for the effect of comorbidities, since it results in the effect of most significant comorbidities, namely those that resolve in death from other causes than cancer. Only the SEER-Medicare database allows the concomitant use of comorbidities and OCM. However, it only holds a fraction (approximately, 30%) of the SEER database population. Consequently, SEER-Medicare- derived observations may not allow sufficient sample size for statistically valid comparisons of small subgroups such as Hispanic/Latinos [25].

Absence of earlier cancer control outcomes, such as biochemical recurrence, progression-free survival or metastatic progression may also be criticized. However, these end points are clearly not as definitive and not as established as the ultimate end point of CSM which is only trumped by overall mortality (OM). However, OM cannot be applied as a valid metric in the context of localized prostate cancer, since a much larger proportion of high-risk localized prostate cancer patients eventually die of other causes than of prostate cancer itself. In consequence, in high-risk localized prostate cancer patients, CSM may be interpreted as the cancer control gold standard and all earlier cancer control end points may be interpreted as its surrogates [20, 26]. Finally, lack of information on subsequent therapies after prostate cancer recurrence may be considered as a limitation. However, this limitation equally applies to all RP and EBRT. In consequence, it represents a non-differential source of potential bias. Non-differential biases are known to have no or marginal effect on the end point of interest.

## Conclusions

Without the use of strictest adjustment for population differences, NCCN high-risk Hispanic/Latino prostate cancer patients appear to benefit more of RP vs EBRT. However, after strictest adjustment for baseline patient and cancer characteristics between the RP and EBRT cohorts, the apparent CSM benefit of RP is no longer statistically significant. In consequence, in Hispanic/Latino NCCN high-risk patients, either treatment modality results in similar CSM outcome.

## Data Availability

All data generated for this analysis were from the SEER database. The code for the analyses will be made available upon request.
